# Ventricular Fibrillation during Optical Coherence Tomography

**DOI:** 10.3390/jcdd11070200

**Published:** 2024-06-29

**Authors:** Paula Vela Martín, Carlos Arellano Serrano, Álvaro Lorente Ros, Juan Francisco Oteo, Arturo Garcia-Touchard

**Affiliations:** 1 Department of Cardiology, University Hospital Puerta de Hierro Majadahonda, 28220 Madrid, Spain; paulavelamartin@gmail.com (P.V.M.); jf.oteo@gmail.com (J.F.O.); 2 Biomedical Research Foundation of University Hospital Puerta de Hierro Majadahonda (FIBHUPH), 28222 Madrid, Spain

**Keywords:** optical coherence tomography, intracoronary imaging, ventricular fibrillation, myocardial ischemia

## Abstract

Optical Coherence Tomography (OCT) imaging is a valuable tool for complex coronary interventions. While complications are rare, the occurrence of ventricular arrhythmias during its use is one of the most feared. Unfortunately, the mechanism by which these arrhythmias arise remains unclear. We describe the case of a patient under continuous electrocardiographic (ECG) monitoring who experienced ventricular fibrillation during an OCT procedure. A detailed analysis of the ECG event sequence was performed, from the administration of the contrast medium to the onset of ventricular fibrillation. Utilizing the collected data, we examined potential precipitating factors based on the observed alterations in the electrocardiogram. A comprehensive understanding of the mechanisms underlying these arrhythmias is crucial for the development of preventive measures that avoid such incidents in the future.

## 1. Introduction

Recent studies have demonstrated the benefits of performing complex percutaneous coronary interventions (PCIs) with the support of intracoronary imaging techniques, and international clinical guidelines recommend their use [1,2]. Optical Coherence Tomography (OCT) uses near-infrared light to obtain in vivo images with high spatial resolution, enabling detailed examination of the interior of coronary vessels. This makes it an invaluable tool for the diagnosis, planning and optimization of PCI in complex scenarios. While complications are rare, the occurrence of ventricular arrhythmias during its use is one of the most feared. Unfortunately, the mechanism by which these arrhythmias arise remains unclear. Understanding the mechanism of their occurrence is essential for prevention. Here, we discuss a case of OCT-guided PCI during which ventricular fibrillation occurred.

## 2. Detailed Case Description

**Figure 1:**
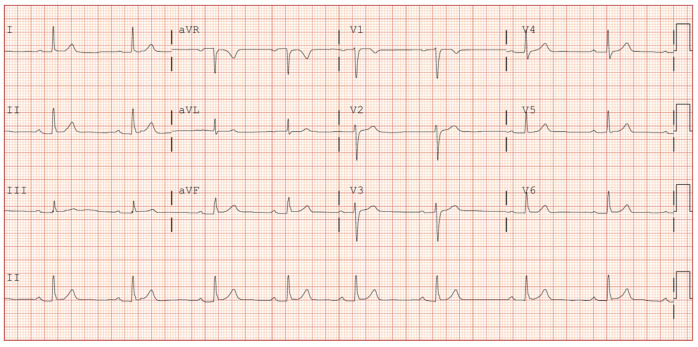
ECG: sinus rhythm at 60 beats per minute, first-degree atrioventricular block (PR 231 ms), narrow QRS and no repolarization alterations. QTc in range.

**Figure 2:**
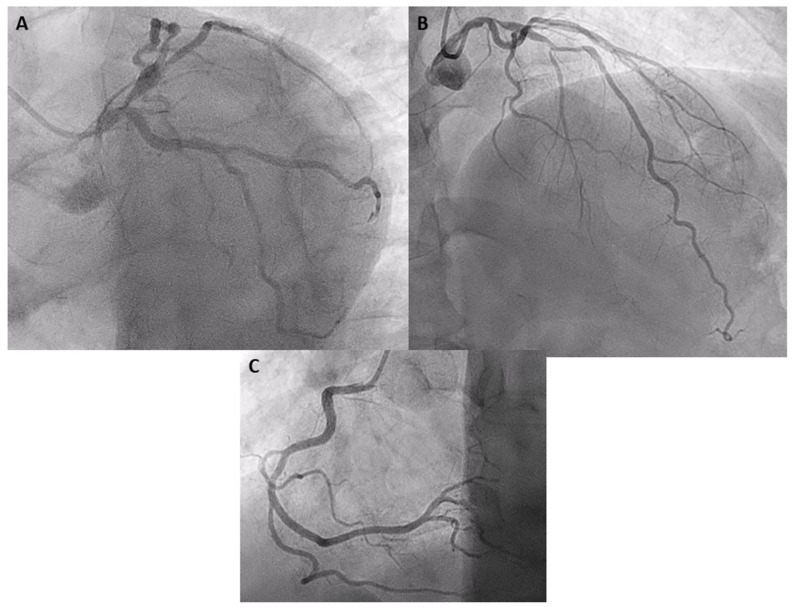
Coronary angiography: (**A**) severe stenosis in the ostial segment of the left anterior descending artery; (**B**) intermediate stenosis in its middle segment; (**C**) dominant right coronary artery without significant stenosis.

A 60-year-old male, current smoker and dyslipidemic, was admitted to the emergency room due to chest pain. He referred to several episodes of chest pain on slight exertion in the last 48 h. His vitals revealed a temperature of 36.5 °C, a heart rate of 55 beats per minute, blood pressure of 130/78 mmHg and oxygen saturation of 99% on room air. A physical examination did not reveal either cardiac murmurs or signs of congestive heart failure. The initial ECG showed sinus rhythm with first-degree atrioventricular block, without repolarization alterations (Figure 1). The transthoracic echocardiogram showed a normal left-ventricular ejection fraction without segmental alterations of contractility and no valvular disease. Initial laboratory reports revealed a slight elevation of cardiac damage markers (ultrahigh-sensitivity troponin I of 158 ng/L [reference range level, 0.0–54.0 ng/L]), with the rest of the analytical parameters in the normal range. With the diagnosis of non-ST-elevation acute coronary syndrome, 300 mg of acetylsalicylic acid and 180 mg of Ticagrelor were administered and the patient was admitted to Cardiology with telemetry. The following morning, the patient underwent a diagnostic coronary angiography via the right radial artery, which revealed a critical stenosis in the ostium of the left anterior descending artery (LAD) and an intermediate stenosis in its middle segment, with diffuse disease between both segments (TIMI III flow). No other significant stenosis was observed in the rest of the coronary vessels (Figure 2).

The patient was informed of the findings and the various treatment options. It was decided to perform PCI in the same procedure guided by intracoronary OCT imaging to assess the anatomy, take measurements and evaluate the possible involvement of the distal left main coronary artery (LMA) as well as the ostium of the LAD and circumflex artery (CX).

**Figure 3:**
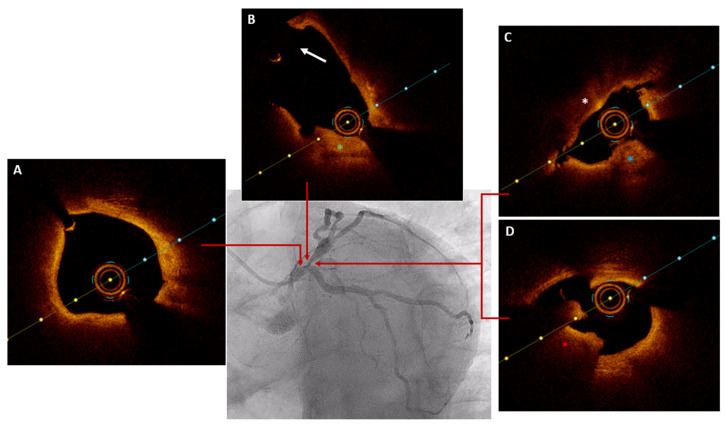
Baseline OCT: (**A**) the distal segment of the left main coronary artery exhibits a lipid plaque that does not cause significant stenosis; (**B**) the left anterior descending artery–circumflex artery bifurcation shows a fibro-lipid plaque (indicated by the green asterisk) affecting the ostium of the left anterior descending artery, without compromising the ostium of the circumflex artery (indicated by the arrow); (**C**) critical stenosis is observed at the ostium of the left anterior descending artery due to a plaque containing both calcium (blue asterisk) and lipids (white asterisk); (**D**) red thrombus is also observed over a ruptured plaque at the ostium of the left anterior descending artery (red asterisk).

Intravenous unfractionated heparin (100 U per kg) was administered. The LMA was catheterized with a 6F 4 EBU guiding catheter, then a Sion guidewire was advanced to the distal segment of the LAD, and a Sion blue guidewire was advanced to the distal segment of the CX. Subsequently, the ostial stenosis of the LAD was dilated using a semi-compliant balloon with a diameter of 2.5 mm. The OCT catheter was then advanced to the mid-distal segment of the LAD, and a first pullback was performed by injecting 16 mL of contrast (4 mL/s for 4 s) without achieving clear intracoronary images. Subsequently, a second pullback was performed, and 29 mL of contrast (5 mL/s for 6 s) were injected through the guide catheter, acquiring optimal images (Figure 3/Video S1/Video S2).

**Figure 4:**
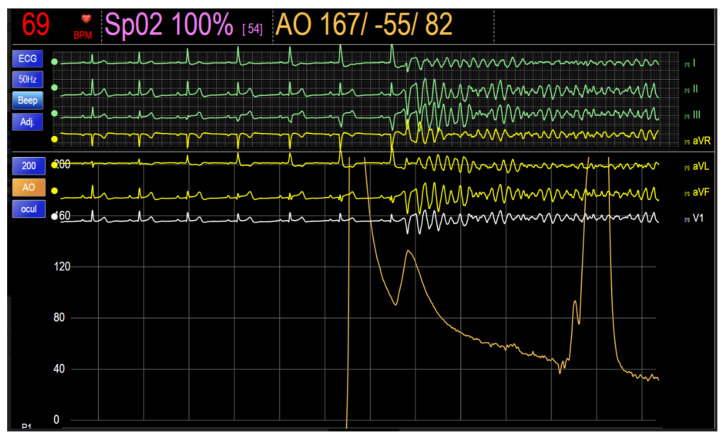
Telemetry during the second Optical Coherence Tomography captures the final moment of contrast injection into the left main coronary artery, explaining the absence of a pulse curve. Subsequently, the pulse wave reappears, followed by a sudden drop in pressure coinciding with the episode of ventricular fibrillation.

Immediately after completing the injection, the patient experienced ventricular fibrillation (VF) (Figure 4), requiring external defibrillation with 360 J, which successfully restored sinus rhythm. The patient recovered promptly. Based on the OCT images obtained from the prior OCT, we proceed with the deployment of a 3.5/18 mm sirolimus-eluting stent from the proximal LAD to the ostium of the LMA, followed by postdilatation of the proximal segment with a 4.5 mm non-compliant balloon at 16 atm. Additionally, the stent struts were opened towards the ostium of the CX using a 2.5 mm non-compliant balloon, concluding with a LAD-CX kissing balloon inflation. Finally, a 2.75/20 mm sirolimus-eluting stent was implanted at 20 atm in the intermediate stenosis of the middle segment of the LAD, which had a minimum luminal area of 2.1 mm^2^.

**Figure 5:**
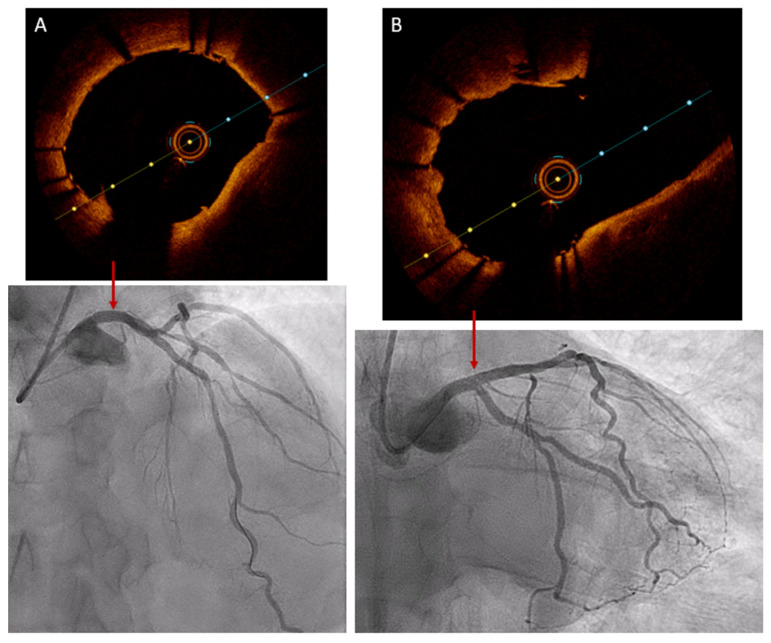
Final result of the percutaneous coronary intervention. The coronary angiography shows stenting in the middle segment of the left anterior descending artery (LAD) and in the left main coronary artery-proximal segment of the LAD. The final OCT shows (**A**) proper expansion and apposition of the stent in the distal segment of the left main coronary artery and (**B**) wide opening of stent struts at the ostium of the circumflex artery.

A new OCT, injecting 15 mL of contrast (4 mL/s for 4 s), revealed adequate apposition and expansion of the LMA stent, with no evidence of distal or proximal dissection, and it also confirmed the correct opening of the stent struts at the ostium of the CX (Figure 5/Video S3). The patient did not experience any further ventricular arrhythmias during this last OCT.

During admission, the patient progressed without complications and was discharged 24 h after catheterization without any further events.

## 3. Discussion

OCT has been evaluated for the assessment of intermediate stenosis [3,4]. Several studies have recently been published demonstrating the benefit of performing complex PCI with the support of various intracoronary imaging techniques. The multicenter OCTOBER trial showed that using OCT during PCI of bifurcation lesions reduces adverse cardiovascular events at 2-year follow-up compared to PCI performed with angiography alone [5]. While intravascular ultrasound (IVUS) has been the traditional method for evaluating LMA lesions, the OCTOBER trial results support the emerging role of OCT as a valuable tool for the assessment of this lesions, as nearly 20% of the patients had a bifurcation lesion involving the LMA [5].

The OCT catheter captures high-resolution images of the inside of the artery by emitting a beam of light [6]. Contrary to IVUS, OCT requires the injection of intracoronary contrast to displace the blood in order to obtain clear images of the vessel. The amount of contrast needed depends on the diameter and length of the segment to be studied, as well as the speed of blood flow. Therefore, there is no fixed guideline for the amount of contrast to be administered. In general, it is recommended to inject 15–20 mL at a rate of 4 mL/second to acquire a pullback in the left coronary tree [6]. However, frequently, this amount of contrast is inadequate to displace the blood from the vessel, resulting in artifacts and making image interpretation challenging. As a result, it is often necessary to repeat acquisitions and substantially increase the final total amount of contrast administered, as occurred in our case, where we had to administer 29 mL to adequately characterize the disease from the middle segment of the LAD to the LMA (55 mm in length).

One of the main concerns about OCT is the higher use of contrast, with the consequent risk of deterioration of renal function. However, in the OPINION trial, which compared OCT-guided PCI with IVUS-guided PCI, although the total amount of contrast was higher in the OCT group (400 patients), none of them suffered from contrast-induced nephropathy [7]. Moreover, in the OCTOBER trial, only one patient in the OCT group suffered acute deterioration of renal function after PCI [5].

From an electrical standpoint, infrequent effects have been described, such as T-wave inversion, ST-segment depression or J-point elevation [8,9]. Complex ventricular arrhythmias are exceptionally rare, for example, in the OCTOBER trial, only three cases of ventricular tachycardia (VT) or VF were reported among the 600 patients who underwent PCI with OCT, without describing the mechanisms that triggered them [5].

In our case, the electrocardiographic recording during contrast injection revealed a progressive ST-segment elevation and a progressive widening of the QRS complex as the contrast was injected into the LMA. Subsequently, a short-coupled extrasystole triggered polymorphic VT that rapidly degenerated into VF (Figure 4), as described by Terada et al. in all their patients [10]. ST elevation and QRS prolongation are described as markers of myocardial ischemia during PCI, with QRS widening being an even more sensitive marker than ST segment changes. This alteration is more pronounced when proximal and middle segments of the main arteries are occluded [11]. The underlying proarrhythmic mechanism of this R-on-T phenomenon has been described in the context of bradycardia, hypoxia, certain drug use and the presence of hydroelectrolyte disturbances [12].

Myocardial ischemia could potentially be attributed to other causes reported in the literature, such as coronary artery spasm, deep intubation of the guiding catheter, air embolization or even dissection/plaque rupture with coronary occlusion during OCT pullback. However, all these possibilities were excluded by the simultaneous angiography done during OCT pullback (Video S2) and in subsequent OCT analysis (Video S3).

Therefore, we believe that the primary cause of the arrhythmia in our patient was myocardial ischemia, which may be attributed to the significant volume of contrast utilized during the second pullback. The posterior R-on-T phenomenon was a key factor in precipitating the subsequent VF.

After defibrillation, the procedure was successfully completed, and a post-PCI control OCT was performed. As we only intended to assess the treated segment in LAD and LMCA (with a total length of 30 mm), we only used half the initial contrast volume, without reproducing any ventricular arrhythmia.

## 4. Conclusions

To the best of our knowledge, this is the first reported case of VF during OCT preceded by widening of the QRS complex and ST elevation, suggesting myocardial ischemia as the mechanism favoring VF. With high probability, the amount of contrast administered in the second pullback was the determinant of VF. For this reason, it is recommended to adjust the volume of contrast injected during OCT acquisition, seeking in each case the balance between image quality and contrast volume. Possible electrical changes in telemetry in the first seconds after OCT should be monitored closely, and a defibrillator should always be available so that rapid action can be taken if ventricular arrhythmia occurs.

## Data Availability

Please note that no data set was generated due to the nature of this publication.

## Supplementary Materials

The following supporting information can be downloaded at: https://www.mdpi.com/article/10.3390/jcdd11070200/s1, Video S1: baseline OCT. Video S2: angiography during baseline OCT. Video S3: Final OCT.
